# TIGIT in Lung Cancer: Potential Theranostic Implications

**DOI:** 10.3390/life13041050

**Published:** 2023-04-19

**Authors:** Carlo Pescia, Giuditta Pini, Edoardo Olmeda, Stefano Ferrero, Gianluca Lopez

**Affiliations:** 1 Pathology Unit, Fondazione IRCCS Ca’ Granda—Ospedale Maggiore Policlinico, Via Francesco Sforza 35, 20122 Milan, Italy; 2 Department of Biomedical, Surgical and Dental Sciences, University of Milan, Via Festa del Perdono 7, 20122 Milan, Italy

**Keywords:** TIGIT, PD-L1, PD-1, prognostic, predictive, theranostic, lung cancer, immune-checkpoint

## Abstract

TIGIT (T cell immunoreceptor with Ig and ITIM domains) is a co-inhibitory receptor expressed on various immune cells, including T cells, NK cells, and dendritic cells. TIGIT interacts with different ligands, such as CD155 and CD112, which are highly expressed on cancer cells, leading to the suppression of immune responses. Recent studies have highlighted the importance of TIGIT in regulating immune cell function in the tumor microenvironment and its role as a potential therapeutic target, especially in the field of lung cancer. However, the role of TIGIT in cancer development and progression remains controversial, particularly regarding the relevance of its expression both in the tumor microenvironment and on tumor cells, with prognostic and predictive implications that remain to date essentially undisclosed. Here, we provide a review of the recent advances in TIGIT-blockade in lung cancer, and also insights on TIGIT relevance as an immunohistochemical biomarker and its possible theranostic implications.

## 1. Introduction

Human neoplasms avoid immune system detection through a variety of immunological escape mechanisms [1]. Tumor cells can decrease T-cell signaling by downregulating the activity of stimulatory receptors while increasing the activity of inhibitory immunoreceptors [2]; for example, they can reduce TCR-mediated stimulatory signaling by downregulating surface MHC-I levels [3], or they may tune up PD-1-mediated inhibitory signaling by increasing PD-L1 surface expression [4]. The hypothesis that inhibiting the activation of inhibitory immunoreceptors might rejuvenate immune cell antitumor action has been shown experimentally and has been successfully applied in the clinical setting for the treatment of various forms of advanced-stage cancers [5,6]. Targeting ligands involved in those interactions with monoclonal antibodies (mAb) has proven to be effective in animal and human tumor models, and immune checkpoint blockade (ICB) with anti-PD-L1, anti-PD-1, or both mAbs is currently regarded as standard therapy for many advanced stage solid malignancies. Moreover, several additional co-inhibitory receptor–ligand interactions, aside from the PD-1/PD-L1 axis, have been described, which can either directly or indirectly suppress the anti-tumor function of CD8+ T cells in the tumor microenvironment. These co-inhibitory receptors include T cell immunoglobulin mucin domain 3 (TIM3) [7], lymphocyte-activation gene 3 (LAG3) [8], cytotoxic T-lymphocyte associated protein 4 (CTLA-4) [9], and T cell immunoreceptor with Ig and ITIM domains (TIGIT) [1]. Interestingly, many lines of evidence suggest that TIGIT is important in reducing adaptive and innate immunity towards malignancies, and anti-TIGIT mAbs have shown promising results in the field of lung cancer [1,10,11]. Specifically, the synergy between TIGIT and the PD-1/PD-L1 axis is being exploited in several clinical trials in which both mechanisms are targeted together, with promising results.

**Figure 1:**
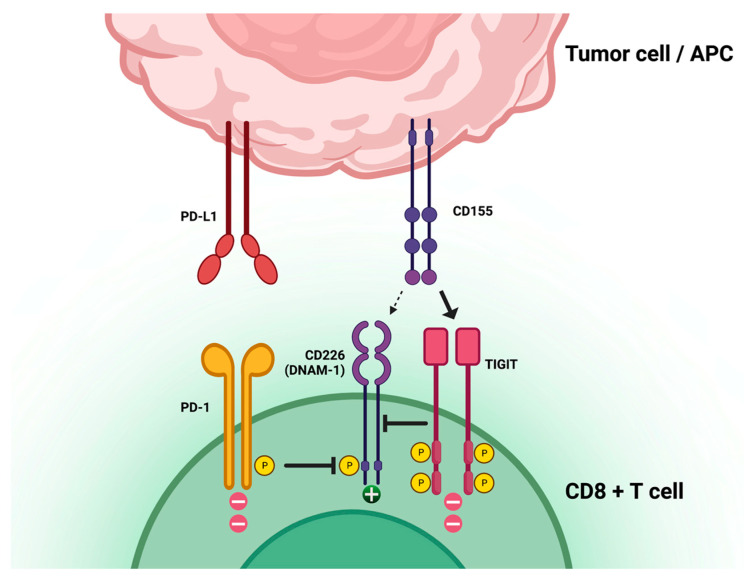
T-cell immunoreceptor with immunoglobulin and immunoreceptor tyrosine-based inhibitory motif domains (TIGIT) location, function, and relation to other immune-checkpoint axes. Created with BioRender.com, accessed on 28 March 2023.

TIGIT is a T-cell receptor involved in limiting T-cell function and adaptive immune responses in the context of cancer immune evasion mechanisms. TIGIT is mostly expressed in T cells and natural killer (NK) cells and has three ligands: CD155, CD112, and CD113. When CD155 is highly expressed on tumor cells, it binds TIGIT and promotes direct and indirect downregulation of T-cell response (Figure 1). The TIGIT/CD155 axis has been shown to play a role in the immune escape and cancer progression of pancreatic cancer [12], ovarian cancer [13], breast cancer [14], and gastric cancer [15]. The interaction of TIGIT with its ligands results in the recruitment of the SHP-1 and SHP-2 phosphatases to the immunoreceptor tyrosine-based inhibition motifs (ITIMs) present in the cytoplasmic domain of TIGIT, leading to the dephosphorylation of downstream signaling molecules and resulting in the inhibition of T-cell activation and proliferation. Additionally, TIGIT can compete with the co-stimulatory receptor CD226 (DNAM-1) in binding CD155 and CD112. CD226 is involved in the activation of T and NK cells, and its engagement with CD155 and CD112 leads to increased cytotoxicity and cytokine production. The competition between TIGIT and CD226 can therefore result in the suppression of immune responses. Moreover, TIGIT has been shown to regulate immune cell metabolism, suppressing glucose uptake and glycolysis in T cells through the inhibition of the Akt-mTOR pathway [15,16,17,18,19]. An association between TIGIT expression and poor survival was identified in multiple malignancies, although with controversial results [20].

In this review, we highlight the current knowledge about TIGIT as a molecular target for lung cancer treatment across all current clinical trials employing anti-TIGIT mAbs; furthermore, we examine the role of TIGIT as a prognostic and predictive biomarker in human cancer, with a focus on immunohistochemistry and its possible prognostic, predictive, and overall theranostic applications on lung cancer.

## 2. Materials and Methods

The online database clinicaltrials.gov was accessed to retrieve the current clinical trials utilizing anti-TIGIT strategies in lung cancer. All 32 results are discussed in Section 3.1. The online database PubMed was accessed to retrieve all current published literature regarding TIGIT as an immunohistochemical biomarker with prognostic and/or predictive value in human cancer. Search keywords included a combination of “TIGIT” and the following: “immunohistochemistry”, “prognostic”, “predictive”, and “biomarker”. A total of 656 articles were identified; 624 were excluded due to lack of relevance and/or discussion about TIGIT as an immunohistochemical biomarker. A total of 32 articles remained and are discussed in Section 3.2.

## 3. Discussion

### 3.1. Clinical Trials in Lung Cancer Utilizing TIGIT-Blockade

Several anti-TIGIT monoclonal antibodies of the IgG1 isotype are currently being evaluated in lung cancer clinical trials (Table 1). The potential effectiveness and the safety of TIGIT inhibitors are being explored mostly in combination with other immune-checkpoint inhibitors or chemotherapies, across different development phases and clinical settings (Table 2) [18,21].

The anti-TIGIT mAb tiragolumab has progressed the furthest in clinical trials for the treatment of non-small cell lung carcinoma (NSCLC). Recently, the Phase II CITYSCAPE (NCT03563716) trial evaluated the possible efficacy of combining tiragolumab with anti–PD-1 antibody atezolizumab in the first-line treatment of NSCLC with PD-L1 expression >1%, assessed by means of the tumor proportion score (TPS). Data showed promising results in favor of the combined treatment (atezolizumab + tiragolumab vs. atezolizumab + placebo) with a longer median survival (PFS 5.6 months vs. 3.9 months; HR 0.58, 95%CI: 0.38–0.89) and an improved objective response rate (ORR 31.3 vs. 16.2%). An exploratory analysis revealed that patients with high PD-L1 expression (TPS ≥ 50%) had a 69% reduction in the risk of disease progression or death from the illness with atezolizumab + tiragolumab vs. 24% with atezolizumab + placebo (PFS 16.6 months vs. 4.11 months; HR 0.29, 95%CI: 0.15–0.53) [11,22]. These results suggest that dual inhibition of immunotherapeutic mechanisms may be effective in clinical practice, although the final results and the design of the Phase III trial in PD-L1 + TPS ≥ 50% population (NCT04294810) are still needed. The CITYSCAPE trial also evaluated the prognostic significance of TIGIT expression. Out of 105 assessable patients, 49 (46.7%) were defined as TIGIT-high (with ≥5% expression on tumor-infiltrating immune cells); no significant impact on progression-free survival (PFS) was noted between TIGIT-high and TIGIT-low groups (HR 0.62, 95% CI 0.30–1.32) [11,18,21].

Although the CITYSCAPE trial found that combined treatment improved ORR and PFS in NSCLC patients, the same combination did not provide any benefits in patients with small cell lung carcinoma (SCLC) in the Phase III SKYSCRAPER-02 (NCT04256421), despite being well-tolerated. However, a Phase II study (NCT04308785) is currently investigating atezolizumab ± tiragolumab as consolidation therapy in limited-stage SCLC participants who have not progressed after receiving chemoradiotherapy.

Moreover, tiragolumab is presently being assessed in a non-metastatic NSCLC setting. The ongoing Phase II SKYSCRAPER-06 trial (NCT04619797) is evaluating atezolizumab + pemetrexed and platinum-based chemotherapy with or without tiragolumab in patients with previously untreated advanced non-squamous NSCLC. Meanwhile, the Phase III SKYSCRAPER-03 trial (NCT04513925) compared atezolizumab and tiragolumab versus durvalumab among patients with locally advanced, unresectable stage III NSCLC. A Phase II study (NCT04832854) is currently underway, with the aim of comparing the effects of neoadjuvant and adjuvant tiragolumab + atezolizumab in combination with chemotherapy versus chemotherapy alone in patients with previously untreated locally advanced resectable stage II, IIIA, or select IIIB NSCLC.

Vibostolimab, another anti-TIGIT mAb, is being studied as monotherapy or in combination with pembrolizumab in NSCLC (NCT02964013). In patients with anti-PD-1/PD-L1-naive NSCLC, treatment-related adverse events (TRAEs) were observed in 85% of cases, with pruritus (38%) and hypoalbuminemia (31%) being the most common ones. The ORR was 26%, with responses occurring in both PD-L1-positive and PD-L1-negative tumors. In contrast, among patients with anti-PD-1/PD-L1-refractory NSCLC, 56% of patients receiving monotherapy and 70% of patients receiving combination therapy experienced TRAEs. The most common adverse events reported were rash and fatigue, affecting 21% of patients on monotherapy, and pruritus (36%) and fatigue (24%) in patients treated with combination therapy. The confirmed ORR was only 6% for monotherapy and 3% for combination therapy. Such results highlight that vibostolimab combined with pembrolizumab exhibited favorable tolerance and showed efficacy in the anti-PD-1/PD-L1-naive population, as well as in both patient subgroups with PD-L1 TPS >1% or <1%. However, the anti-tumor effects of vibostolimab alone or in combination with pembrolizumab were limited in the anti-PD-1/PD-L1-refractory population [23]. Moreover, an ongoing Phase III (NCT04738487) trial is assessing pembrolizumab alone and in conjunction with vibostolimab in PD-L1 positive NSCLC patients. The available data on tiragolumab and vibostolimab indicate a need for further clarification on the appropriate setting for dual anti-TIGIT+anti-PD-1/PD-L1 therapy. The results show that higher ORRs were achieved in the anti-PD-1/PD-L1-naïve population, suggesting that administering the combination therapy upfront may be optimal for preventing or delaying the development of immune checkpoint inhibitor (ICI) resistance. Conversely, the ORR was significantly lower among the anti-PD-1/PD-L1-refractory population, highlighting the limitations in treating acquired ICI resistance [24].

Other combinations of anti-TIGIT and anti-PD-L1 or anti-PD-1 have shown promising activity in NSCLC. The Phase II ARC-7 trial (NCT04262856) is currently investigating the combination of the anti-TIGIT domvanalimab and zimberelimab (an anti-PD-1 drug) on PD-L1-positive locally advanced or metastatic NSCLC patients. In addition, a phase II study (NCT 04791839) is evaluating the use of domvanalimab + zimberelimab along with etrumadenant (an adenosine receptor antagonist) in previously treated NSCLC patients [25]. A Phase III (NCT04746924) study is underway to assess the effectiveness of ociperlimab + tislelizumab, as opposed to pembrolizumab, in previously untreated patients with advanced NSCLC and PD-L1 tumor cell ≥ 50% expression [26].

In a Phase I trial (NCT03119428), the anti-TIGIT antibody etigilimab was tested alone or in combination with the anti-PD-1 antibody nivolumab in patients with locally advanced or metastatic solid tumors. The most reported adverse events in Phase Ia and Ib were rashes, nausea, fatigue, and a decreased appetite. Six patients experienced severe TRAEs, while a few patients showed stable disease or partial response. The median PFS was approximately 56 days in Phase Ia and 57 days in Phase Ib. The study also identified evidence of etiligimab’s dose-dependent immune modulation through flow cytometry and PCR biomarker correlative analyses, including the activation of immune T-cell subpopulations and the decrease in peripheral Tregs [27]. Although promising results were documented in terms of safety and antitumor activity during Phase Ia, Phase Ib was not carried on due to the sponsor’s decision [28,29].

Many other human anti-TIGIT mAbs are currently being tested in Phase I/II clinical trials in combination with PD-1/PD-L1 blockade or chemotherapies for the treatment of advanced lung cancer. Preliminary results show that a combination of these agents with PD-1/PD-L1 inhibition in NSCLC leads to higher response rates compared with PD-1/PD-L1 inhibition alone, possibly due to the synergistic mechanisms of action, including the increased activation of NK cells and CD8+ TILs [30,31,32]. Further studies will be necessary to determine the proper sequence of specific therapy regimens of these mAbs, and identify which patients would benefit from early chemotherapy combinations [33]. Additional research is required to comprehensively understand the approaches to enhance immune regulation in SCLC patients, possibly prioritizing the investigation of the molecular subtypes.

Anti-TIGIT strategies are being investigated in other human malignancies aside from lung cancer, with promising initial results [28]. In the future, anti-TIGIT therapies could become a standard-of-care; identifying an inexpensive and easily accessible predictive biomarker would aid greatly in patients’ stratification and management, with overall improved patient care.

**Table 1:** Anti-TIGIT antibodies currently in lung cancer clinical trials.

Agent	Isotype	Company/Sponsor	Clinical Phase	Identifier
Tiragolumab	Fully human IgG1/kappa	Roche	II/III	NCT03563716 [11,22,34] NCT04294810 NCT04513925 NCT04619797 NCT04832854 NCT04958811 NCT05034055 NCT03977467 NCT04308785 NCT04256421
Vibostolimab	Fully human IgG1	Merck Sharp and Dohme	I//II/III	NCT04165798 NCT04725188 NCT04738487 NCT02964013 [23] NCT04165070
Ociperlimab	Humanized IgG1	BeiGene	II/III	NCT04746924 [26] NCT04866017 NCT04952597 NCT05014815
Domvanalimab	Fully human IgG1	Arcus Biosciences	I//II/III	NCT04262856 [25] NCT04736173 NCT04791839 NCT03628677
EOS-448	Fully human IgG1	iTeos Therapeutics	I/II	NCT05060432 NCT03739710
SEA-TGT	Nonfucosylated human IgG1	Seagen Inc	Ib/II	NCT04585815
IBI939	Fully human	Innovent Biologics	I	NCT04672369 NCT04672356
AZD2936	Humanized IgG1	AstraZeneca	I/II	NCT04995523
HLX301	Recombinant Humanized IgG1	Shanghai Henlius Biotech	I/II	NCT05102214
Etigilimab	Humanized IgG1	OncoMed Pharmaceuticals	I	NCT03119428 [28]

**Table 2:** Ongoing trials with new immune checkpoints targets in lung cancer. Abbreviations: EGFR = epidermal growth factor receptor; ALK = anaplastic lymphoma kinase; NSCLC = non-small cell lung cancer; PD-L1 = programmed cell death ligand 1; cCRT = concurrent chemoradiotherapy; ICI = immune checkpoint inhibitor; SBRT = stereotactic body radiotherapy; PD-1 = programmed death 1; SCLC = small cell lung cancer; SCCHN = squamous cell carcinoma of the head and neck; RCC = renal cell carcinoma; CRT = chemoradiotherapy; CT = chemotherapy; SoC = standard of care; LC = lung cancer.

Trial ID	References	Status	Therapy Regimen	Setting	Trial Phase and Type	N
*CITYSCAPE* NCT03563716	Cho et al., Lancet Oncol 2022 [11]; Bendell et al., Cancer Res 2020 [22]	Active, non recruiting	Tiragolumab + atezolizumab vs. placebo + atezolizumab	*EGFR/ALK* wild-type NSCLC with PD-L1 ≥ 1%	Phase II, randomised, double-blinded, placebo-controlled	67 vs. 68
*SKYSCRAPER-01* NCT04294810	-	Recruiting	Tiragolumab + atezolizumab vs. placebo + atezolizumab	Untreated locally advanced unresectable or metastatic NSCLC with PD-L1 ≥ 50%	Phase III, randomized, double-blinded, placebo-controlled	Estimated 660
*SKYSCRAPER-03* NCT04513925	-	Recruiting	Tiragolumab + atezolizumab vs. Durvalumab	Locally advanced, unresectable stage III NSCLC, after cCRT	Phase III, randomized, open-label	Estimated 800
*SKYSCRAPER-06* NCT04619797	-	Recruiting	Tiragolumab + atezolizumab + pemetrexed + carboplatin or cisplatin vs. pembrolizumab pemetrexed + carboplatin or cisplatin	Previously untreated advanced non-squamous NSCLC	Phase II, randomized, double-blinded, placebo-controlled	Estimated 540
NCT04832854	-	Recruiting	Neoadjuvant and adjuvant tiragolumab + atezolizumab, with or without platinum-based chemotherapy	Resectable stage II, IIIA, or select III B NSCLC	Phase II, multicenter, open-label	Estimated 82
NCT04958811	-	Recruiting	Tiragolumab with atezolizumab + bevacizumab	ICI pretreated, PD-L1+, non-squamous NSCLC	Phase II, multi-cohort, open-label	Estimated 42
*SKYROCKET* NCT05034055	-	Not yet recruiting	SBRT followed by atezolizumab/tiragolumab	Treatment naïve metastatic NSCLC	Phase II, open-label	Estimated 45
NCT03977467	-	Recruiting	Atezolizumab + tiragolumab	NSCLC or advanced solid tumors with prior PD-1 inhibitor treatment	Phase II, open-label	Estimated 80
NCT04308785	-	Active, non recruiting	Atezolizumab ± tiragolumab as consolidation therapy	Limited stage SCLCs who have not progressed to chemoradiotherapy	phase II, randomized, double-blinded, placebo-controlled	24
*SKYSCRAPER-02* NCT04256421	-	Active, non recruiting	Atezolizumab + carboplatin and etoposide ± tiragolumab	Untreated extensive stage SCLC	Phase III, randomized, double-blinded, placebo-controlled	490
NCT02964013	Niu et al., Ann Oncol 2022 [23]	Active, non recruiting	Vibostolimab vs. vibostolimab + pembrolizumab vs. vibostolimab + pembrolizumab	Anti-PD-1/PD-L1-refractory NSCLC	Phase I, multicenter, open-label	34 vs. 33 vs. 39
				Anti-PD-1/PD-L1-refractory NSCLC		
				Anti-PD-1/PD-L1-naive NSCLC		
*KEYMAKER-U01* NCT04165798	-	Recruiting	Vibostolimab + pembrolizumab + chemotherapy vs. vibostolimab + pembrolizumab vs. vibostolimab + pembrolizumab	Treatment naive NSCLC	Phase II, multi-cohort	Estimated 260
				Treatment naïve PD-L1 positive NSCLC		
				NSCLC previously treated with anti-PD-L1 NSCLC		
NCT04738487	-	Recruiting	Pembrolizumab/vibostolimab coformulation (MK-7684°) vs. pembrolizumab	NSCLC with PD-L1 ≥ 1%	Phase III, multicenter, randomized, double-blinded	Estimated 1246
NCT04165070	-	Recruiting	Pembrolizumab + carboplatin + paclitaxel vs. vibostolimab	Treatment naïve advanced NSCLC	Phase II, open-label	Estimated 360
NCT04725188	-	Active, non recruiting	Pembrolizumab/vibostolimab coformulation (MK-7684A) or pembrolizumab/vibostolimab coformulation (MK-7684A) + docetaxel vs. docetaxel	ICI and platinum chemotherapy pretreated	Phase II, multicenter, randomized	Estimated 240
*ARC-7* NCT04262856	Catalano et al., Cancers (Basel). 2022 [25]	Active, non recruiting	Domvanalimab + zimberelimab (A2BR antagonist) vs. zimberelimab vs domvanalimab + zimberelimab + etrumadenant (dual adenosine A2a/A2b receptor antagonist)	NSCLC with PD-L1 expression of ≥ 50%	Phase II, open-label, randomized	Estimated 150
*ARC-10* NCT04736173	-	Recruiting	Domvanalimab + zimberelimab vs. zimberelimab vs. chemotherapy	Locally advanced or metastatic NSCLC, with PD-L1 ≥ 1%	Phase III, open-label, randomized	Estimated 625
NCT04791839	-	Recruiting	Domvanalimab + zimberelimab (anti-PD-1) + etrumadenant (A2R inhibitor)	ICI pretreated, NSCLC with PD-L1 ≥ 1%	Phase II, open-label	Estimated 30
NCT03628677	-	Active, non recruiting	Domvanalimab ± AB122 (anti PD-1)	Advanced or metastatic NSCLC, SCCHN, RCC, breast cancer, colorectal cancer, melanoma, bladder cancer, ovarian cancer, endometrial cancer, Merkel cell carcinoma, or gastroesophageal cancer	Phase I, open-label	75
*AdvanTIG-302* NCT04746924	Socinski et al., Clin Oncol. 2021 [26]	Recruiting	Ociperlimab + tislelizumab vs. pembrolizumab + placebo vs. tislelizumab + placebo	NSCLC and PD-L1 tumor cell ≥ 50% expression	Phase III multicenter, randomized, double-blind	Estimated 660
NCT04866017	-	Recruiting	Ociperlimab + tislelizumab + cCRT → ociperlimab + tislelizumab or tislelizumab + cCRT → tislelizumab vs. cCRT → durvalumab	Untreated, locally advanced, unresectable NSCLC	Phase III, open-label, randomized	Estimated 900
NCT04952597	-	Active, non recruiting	Ociperlimab + tislelizumab + CRT	Untreated, limited stage SCLC	Phase II, multicenter, randomized, open-label	126
NCT05014815	-	Recruiting	Ociperlimab and tislelizumab + CT	Untreated locally advanced, unresectable, or metastatic	Phase II, randomized	Estimated 270
NCT05060432	-	Recruiting	EOS-448 + SoC and/or investigational therapies	Advanced NSCLC	Phase I/II, multicenter, open-label	Estimated 376
NCT03739710	-	Recruiting	Feladilimab, ipilimumab (anti-CTLA-4), EOS-448, dostarlimab (various combination) vs. SoC	Relapsed/refractory advanced NSCLC	Phase II, open-label, randomized	Estimated 185
NCT04672369	-	Active, non recruiting	IBI939 + sintilimab (anti-PD-1)	Advanced LC	Phase I, open-label, randomized	Estimated 42
NCT04672356	-	Active, non recruiting	IBI939 + sintilimab	Advanced LC	Phase I, open-label	Estimated 20
NCT04585815	-	Active, non recruiting	SEA-TGT + sasanlimab (anti-PD-1) + Axitinib	Advanced NSCLC	Phase Ib/II, open-label	23
*ARTEMIDE-01* NCT04995523	-	Recruiting	AZD2936 (anti-TIGIT/anti-PD-1 bispecific antibody)	Locally advanced or metastatic NSCLC	Phase I/II, open-label	Estimated 192
NCT05102214	-	Recruiting	HLX301 (PDL1/TIGIT bispecific Ab)	Locally advanced or metastatic solid tumors	Phase I/II, open-label	Estimated 150
NCT03119428	Mettu et al., Clin Cancer Res., 2022 [28]	Terminated (Sponsor decision)	Etigilimab ± nivolumab (anti PD-1 mAb)	Advanced or metastatic solid tumors	Phase I, open-label	33

### 3.2. TIGIT as an Immunohistochemical Biomarker: Current Knowledge

Numerous studies have investigated the expression and prognostic significance of TIGIT in various human cancers, including melanoma, NSCLC, hepatocellular carcinoma, thyroid cancer, gastric cancer, and colorectal cancer. These studies have reported varying levels of TIGIT expression in different cancer types and stages, with high TIGIT expression being associated with poor prognosis in some cases and favorable prognosis in others. TIGIT immunohistochemistry (IHC) has been performed with various antibodies for various purposes, mostly in association with genomic, transcriptomic, flow cytometry, and/or fluorescence techniques, with IHC usually serving as a validation tool for TIGIT protein expression. Different scoring systems were adopted for TIGIT IHC evaluation, depending on the focus either on the tumor microenvironment or cancer cells (Table 3).

Predictably, TIGIT expression has mostly been found in CD3+ tumor-infiltrating lymphocytes (TILs) and peritumoral lymphocytic infiltrates, given its physiological role, highlighting an “exhausted” T-cell phenotype in a consistent proportion of cancer microenvironments. Moreover, TIGIT expression has been found to positively correlate with PD-1 and PD-L1 density in the tumor microenvironment (TME), highlighting the synergy between the two immune-checkpoint axes, as seen in lung squamous cell carcinoma, lung adenocarcinoma, and melanoma [35,36,37]. These findings justify and explain the success of TIGIT immunotherapy in PD-L1+ non-small cell lung carcinoma [11,38]. TIGIT expression was also documented on tumor cells, especially in cutaneous melanoma [39], choroidal melanoma [40], thyroid cancer [41], undifferentiated pleomorphic sarcoma [42], lung adenocarcinoma [43], and esophageal cancer [44]. An interesting study has also demonstrated TIGIT expression on CD20+ TILs in gastric cancer [45], where cases with higher TIGIT+CD20+ infiltrates exhibited a worse prognosis.

The majority of studies investigating TIGIT expression in TME across different malignancies have shown its negative impact on overall survival, progression-free survival, disease-free survival, recurrence-free survival, or its association with increased hazard for metastatic disease (Table 3). However, results are conflicting, and several studies have failed to prove a prognostic role for TIGIT expression, specifically in esophageal cancer [46], medullary thyroid carcinoma [47], NSCLC [34,48,49], and SCLC [50]. In contrast, other authors have reported a positive prognostic impact of TIGIT-enriched TME on survival, as seen in oral squamous cell carcinoma [51], breast cancer [52], and melanoma [39].

**Table 3:** Publications that explored TIGIT immunohistochemistry in human cancer. (Abbreviations: overall survival, OS; progression-free survival, PFS; recurrence-free survival, RFS; disease-free survival, DFS; tumor-infiltrating lymphocytes, TILs; immunohistochemistry, IHC; high power field, HPF; tumor microenvironment, TME).

Antibody	Publication	Cancer Type	Visualization	Correlations	*p*-Value
Abcam, ab243903 Rabbit monoclonal (BLR047F clone)	Wang, P. et al. [46]	Esophageal cancer	H-score	No difference in 3-year OS between TIGIT+ and TIGIT- cases	0.140
	Xu, X. et al. [53]	Esophageal cancer	Multiplex fluorescence immunohistochemistry	TIGIT expression in TME is positively associated with *AIF1* expression, a differentially expressed gene that negatively impacts on prognosis.	0.013
	Steele, NG. et al. [54]	Pancreatic ductal adenocarcinoma	Multiplex fluorescence immunohistochemistry	Validation at the protein level that CD8+ TILs show enriched TIGIT expression	/
	Liu, Z. et al. [55]	Urothelial carcinoma	Mean number of positive cells extracted from the view of three HPF	TIGIT+ CD8+ cells high infiltration group possessed inferior OS and RFS compared with the TIGIT+ CD8+ cells low infiltration group	0.01
	Liu, Z. et al. [56]	Urothelial carcinoma	Mean number of positive cells extracted from the view of three HPF	PD-1+ cells infiltration had no prognostic value in patients with high TIGIT+ cells infiltration. Patients with high TIGIT expression, irrespectively of the number of PD1+ cells, exhibited poorer prognosis	0.024
	Eichberger, J. et al. [51]	Oral squamous cell carcinoma	Assessment of semiquantitative percentage of TIGIT expression within CD3+ T cells (ranging from 0–100%)	TIGIT expression on CD3+ cells correlates with improved OS	0.025
	Shi, X. et al. [47]	Medullary thyroid carcinoma	Combined positive score (CPS) algorithm, defined as the percentage of positive tumor cells (total/partial membrane staining) and TILs (membrane/cytoplasm staining) relative to the total number of tumor cells, multiplied by 100. Expression was further stratified into low (1 ≤ CPS < 5), moderate (5 ≤ CPS < 20), and strong (CPS ≥ 20).	TIGIT expression had no impact on prognosis	0.448
	Guo, C. et al. [57]	Breast cancer	ImageJ analysis of IHC	TIGIT is significantly upregulated in invasive breast tumor TME compared with normal tissues; this finding is confirmed using IHC	/
	Duan, X. et al. [58]	Hepatocellular carcinoma	Manual counting	TIGIT expression in TILs gradually increased in liver cancer tissues as the degree of tumor cell differentiation changed from high to low	/
	Nakazawa, T. et al. [41]	Thyroid cancer	Semiquantitative evaluation of percentage of positive epithelial cells (0: less than 1%, 1+: 1–49%, and 2+: more than 50%)	Expression in tumor cells was detected in medullary thyroid carcinoma, anaplastic thyroid carcinoma, and poorly differentiated thyroid carcinoma, while it was absent in benign lesions/tumors and differentiated carcinomas. Pleomorphic/giant cell morphology seemed to correlate with TIGIT expression in anaplastic thyroid carcinomas.	<0.05
	Jiang, C. et al. [48]	Non-small cell lung cancer	Inflammatory infiltrates in all the samples were assessed and subclassified semi quantitatively into TIGIT-negative (≤5% stained) or positive (>5% stained)	TIGIT expression in TME had no impact on PFS in patients treated with anti-PD1 therapy	0.092
	Ishihara, S. et al. [42]	Undifferentiated pleomorphic sarcoma	TIGIT expression was considered low when tumor cells did not express TIGIT or showed a very weak immunopositivity despite immune cells showing strongly positive expression	Expression of TIGIT on tumor cells tended to be associated with poorer OS	0.555
	Luo, Y. et al. [59]	Advanced thyroid carcinomas	Combined positive score (CPS) algorithm, defined as the percentage of positive tumor cells (total/partial membrane staining) and TILs (membrane/cytoplasm staining) relative to the total number of tumor cells, multiplied by 100. Expression was further stratified into negative (CPS <1), weak (1 ≤ CPS < 10), moderate (10 ≤ CPS < 30), and strong (CPS ≥30)	TIGIT expression had a negative impact on OS	0.004
	Stålhammar, G. et al. [40]	Choroidal melanoma	Number of TIGIT positive cells per 3 HPF, corresponding to an aggregated area of 0.6 mm^2^	Time dependent hazard for metastasis was significantly increased for patients with a number of TIGIT positive cells/mm^2^ in primary tumor hot spots above the median	0.03
TIGIT XP^®^ #99567 Rabbit monoclonal (E5Y1W clone)	Liu, L. et al. [60]	Cervical cancer	Multiplex fluorescence immunohistochemistry	The number of CD8+TIGIT+ cells in cervical cancer tissues was significantly higher than that in adjacent cancer tissues.	<0.01
	Liu, H. et al. [45]	Gastric cancer	Dual IHC, counting the number of TIGIT+CD20+ B cells in three representative HPFs (×200 amplification), was calculated for each section and the average of the three values was used as the final counting result	High peritumoral TIGIT+CD20+ B cell infiltration was associated with worse - OS - DFS.	< 0.001 0.0252
	Boissière-Michot, F. et al. [52]	Breast cancer	H-score	TIGIT+ cell density in TME tended to be associated with better RFS	0.079
	Yang, Z. et al. [35]	Lung squamous cell carcinoma	The number of TIGIT+ TILs was counted in six HPFs. TIGIT density was defined as high or low using the median count as the cut-off value.	High TIGIT density was correlated with positive PD-L1 expression, high PD-1 density, and high CD8 density. High TIGIT density correlated with worse prognosis.	/ 0.027
	Ducoin, K. et al. [61]	Colorectal cancer	Regions of interest were drawn (tumor glands and peritumoral stroma near the invasive margin). In each region (tumor and stroma), a total number of 5000 cells were counted in the 3 areas per section, and the results are expressed as the mean of the 3 counts	Microsatellite instability correlate with higher expression of TIGIT+CD3+ TILs	0.0131
	Shen, M. et al. [49]	Lung adenocarcinoma	Inflammatory infiltrates in all samples were assessed and subclassified semi quantitatively into TIGIT-negative (≤5% stained) or positive (>5% stained)	TIGIT expression had no impact on - RFS - OS	0.564 0.152
TIGIT antibody Dianova, Hamburg, Germany Rabbit monoclonal (TG1 clone)	Blessin, N.C. et al. [62]	Human cancer TMA	The number of TIGIT+ cells per 0.6 mm tissue spot was manually counted and converted into the density of TIGIT+ cells per square mm	Highest densities of TIGIT+ TILs were found in tumors characterized by high numbers of TILs. In colorectal cancers, expression of TIGIT and PD-1 was considerably higher in T cells located at the invasive margin as compared with T cells in the tumor center, overlapping with PD1 expression	/
	Li, W. et al. [63]	Hodgkin’s lymphoma	Percentage of stained cells in the lymphocytic background (median value 86%)	Highest staining intensities were found in a case of NLPHL; staining intensity of the T-cell rosettes surrounding malignant cells in NLPHL and in LRCHL appeared stronger	/
	Niebel, D. et al. [39]	Melanoma	H-score for cancer cells; TIGIT+ immune cells were assessed as percentage fraction from all cells (TIGIT+ lymphocyte score)	Patients with TIGIT+ lymphocyte scores > 1% had a significant better progression-free survival compared with patients with TIGIT+ lymphocyte scores ≤ 1%. TIGIT was detected also in several melanoma cells	0.010
	Müller, S. et al. [43]	Lung adenocarcinoma	H-score	TIGIT expression was heterogeneous among cancer cells and TILs. TIGIT expression was observed in malignant and not in benign cells, with increasing proportions from pre-malignant to overtly malignant lesions	/
	Scimeca, M. et al. [64]	Prostate adenocarcinoma	TIGIT+ TILs were evaluated with the support of a digital software (Image Viewer, Ventana, Roche) by two blind observers by counting the number of positive prostate cells on 9.42 mm^2^ prostate tissues	No significant differences were observed in TIGIT+ TILs between prostate adenocarcinoma and benign lesions	0.9833
TIGIT antibody Biomatik, Wilmington, DE, USA Rabbit monoclonal (TG1 clone)	Lee, W. J. et al. [37]	Cutaneous melanoma	Staining intensity on TILs was determined on a scale of 0–3, with zero indicating <5%, 1 indicating 5–20%, 2 indicating >20–50%, and 3 indicating >50% of TILs. Cases with a score ≥1 were considered positive.	High TIGIT expression in TILs was associated with deeper Breslow thickness, more vertical growth, higher mitotic counts, higher frequency of lymph node involvement and advanced AJCC stage, higher density of TILs, higher expression of PD-1, and poorer OS and PFS	<0.04 for all parameters
TIGIT Santa Cruz sc-103349	Lucca L.E. et al. [65]	Glioblastoma (GBM) and multiple sclerosis (SM) samples	Immunolabeled cells with a lymphocytic morphology were manually quantified and the counts were averaged. The number of TIGIT+ cells was correlated with the number of CD3+ lymphocytes found in each region of interest.	- The percentage of TIGIT+ T cells was substantially higher in GBM infiltrates compared with MS lesions. - In GBM, the percentage of TIGIT+ T cells was significantly higher in tumor tissue than in perivascular infiltrates	0.04 0.017
	Xu, Y. et al. [50]	Lung small cell carcinoma	Positively stained sections were analyzed using the integrated optical density (IOD) and the areas of staining distribution with NIS-Elements Br version 4.60.00; the mean density was obtained by dividing the IOD value by the area, and an average from 5 representative fields was calculated (magnification, ×400)	TIGIT expression did not impact OS	0.874
TIGIT MYBioSource #MBS20013451, Rabbit polyclonal	Sun, Y. et al. [36]	Lung adenocarcinoma	Inflammatory infiltrates in all the samples were assessed and subclassified semi quantitatively into TIGIT-negative (≤5% stained) or positive (>5% stained)	TIGIT expression positively correlated with PD-1 and PD-L1 and portended worse OS	0.024
TIGIT NBP2-79793, Novus, USA Rabbit monoclonal (TIGIT/3017 clone)	Zhao, K. et al. [44]	Esophageal small cell carcinoma	TIGIT expression was assessed manually and semi-quantitatively in tumor cells as follows: ≤5% staining was considered negative and >5% staining was scored as positive	- TIGIT positivity was higher in tumor tissues than in the matched adjacent tissues. - TIGIT-positive patients had a shorter OS than TIGIT-negative patients - TIGIT-positive cases had lower PFS	<0.001 0.001 0.034
TIGIT Thermo Fisher Scientific, Rabbit monoclonal (MBSA43 clone)	Zhao, J. J. et al. [66]	Esophageal squamous cell carcinoma	Average number of TIGIT+ immune cells was calculated as the final density of each section (cells/mm^2^)	Patients carrying a high number of TIGIT+ TILs (n = 76/154, 49.4%) tended to exhibit a shorter OS Cancers enriched with PD-1+/TIGIT+ TILs demonstrated significantly lower survival rates than patients with PD-1−/TIGIT− TILs	0.045 0.005
TIGIT IHC assay Roche Tissue Diagnostics SP410 antibody	Patil, N. et al. [34]	Non-small cell lung cancer (CITYSCAPE TRIAL)	Evaluating immune cells only, ≤5% staining was considered low and >5% staining was scored as high	No association between high TIGIT expression and PFS	/

Further studies are needed to deepen our knowledge of TIGIT expression, both in the neoplastic microenvironment and in tumor cells, and its substantial correlation with the PD1/PD-L1 axis, particularly in the field of lung cancer, on which most immunotherapy trials are focused. To date, a single clinical trial evaluated the significance of TIGIT IHC as a biomarker, specifically in a prognostic way [11,22,34]. TIGIT immunohistochemical expression is currently not considered a prerequisite for the administration of TIGIT inhibitors, such as tiragolumab in non-small-cell lung cancer, for which PD-L1 positivity is deemed sufficient, and no data exist about the potential role of TIGIT expression as a predictive biomarker for response to anti-TIGIT regimens. Current clinical trials mostly utilize anti-TIGIT regimens as an addition to anti-PD-1/PD-L1 or anti-CTLA-4 inhibition, with few exceptions: a Phase I trial in which vibostolimab is administered to anti-PD-1/PD-L1-refractory NSCLC (NCT02964013), a Phase II trial in which vibostolimab is utilized in treatment naïve advanced NSCLC (NCT04165070), and a terminated Phase I trial which used etigilimab in advanced/metastatic solid malignancies, lung included (NCT03119428). These examples highlight the clinical need for a predictive biomarker of responses to anti-TIGIT therapy regimens, in which IHC could play a significant role in stratifying patients who could benefit most from the therapy and patients in which therapy could be ineffective and unnecessary, paralleling the PD-L1 experience, particularly in the lung. In this view, TIGIT IHC may reveal a theranostic utility, potentially guiding complex therapeutic approaches, and providing novel insights into the complexity of TME.

## 4. Conclusions

The importance of TIGIT as a target for immune-checkpoint inhibition in lung cancer is becoming more and more clear as clinical trials continue to progress and provide results on the therapeutic effectiveness of anti-TIGIT mAbs. As for now, the prognostic value of TIGIT expression in human malignancies, assessed with IHC, is controversial, with different results across different types of human cancer. The predictive role of TIGIT expression is understudied and largely unknown. Although solid, the current assumption that TIGIT inhibition has to rely on PD-1/PD-L1 axis inhibition, on which most clinical trials using anti-TIGIT strategies are based, may be not totally comprehensive; differences could exist between different types of human cancer in relation to the significance of TIGIT expression and its relationship with PD-1/PD-L1, and the relevance of TIGIT expression might have been overlooked, especially in anti-TIGIT monotherapy regimens trials. To investigate whether TIGIT expression in CD8+ TILs in human cancer is predictive to anti-TIGIT therapy could provide insights into a novel and inexpensive tool for patients’ treatment stratification, thus potentially reducing overtreatment and collateral effects.

## Data Availability

Not applicable.
